# Effective Approaches to Fetal Brain Segmentation in MRI and Gestational Age Estimation by Utilizing a Multiview Deep Inception Residual Network and Radiomics

**DOI:** 10.3390/e24121708

**Published:** 2022-11-23

**Authors:** Moona Mazher, Abdul Qayyum, Domenec Puig, Mohamed Abdel-Nasser

**Affiliations:** 1 Departament d’Enginyeria Informatica i Matemátiques, Universitat Rovira i Virgili, 43007 Tarragona, Spain; 2 School of Biomedical Engineering and Imaging Sciences, Kings College London, London SE1 9RT, UK; 3 Electronics and Communication Engineering Section, Electrical Engineering Department, Aswan University, Aswan 81528, Egypt

**Keywords:** multi-view segmentation, fetal brain, fetal age prediction, deep learning, machine learning

## Abstract

To completely comprehend neurodevelopment in healthy and congenitally abnormal fetuses, quantitative analysis of the human fetal brain is essential. This analysis requires the use of automatic multi-tissue fetal brain segmentation techniques. This paper proposes an end-to-end automatic yet effective method for a multi-tissue fetal brain segmentation model called IRMMNET. It includes a inception residual encoder block (EB) and a dense spatial attention (DSAM) block, which facilitate the extraction of multi-scale fetal-brain-tissue-relevant information from multi-view MRI images, enhance the feature reuse, and substantially reduce the number of parameters of the segmentation model. Additionally, we propose three methods for predicting gestational age (GA)—GA prediction by using a 3D autoencoder, GA prediction using radiomics features, and GA prediction using the IRMMNET segmentation model’s encoder. Our experiments were performed on a dataset of 80 pathological and non-pathological magnetic resonance fetal brain volume reconstructions across a range of gestational ages (20 to 33 weeks) that were manually segmented into seven different tissue categories. The results showed that the proposed fetal brain segmentation model achieved a Dice score of 0.791±0.18, outperforming the state-of-the-art methods. The radiomics-based GA prediction methods achieved the best results (RMSE: 1.42). We also demonstrated the generalization capabilities of the proposed methods for tasks such as head and neck tumor segmentation and the prediction of patients’ survival days.

## 1. Introduction

Congenital disorders are some of the leading causes of infant mortality worldwide [1]. Recently, in utero magnetic resonance imaging (MRI) of the fetal brain has emerged as a valuable tool for investigating the neurological development of fetuses with congenital disorders to aid in prenatal planning. Fetal MRI requires clinical and technical expertise and is a challenging imaging modality due to the ability to move freely. T2-weighted single-shot fast spin echo (ssFSE) sequences, such as ultra-fast MRI sequences, can be used to attain information in all planes.

Super-resolution (SR) reconstruction algorithms, including outlier rejection and motion correction strategies [1], can then be applied in order to combine several low-resolution images into a single high-resolution volume that can be used for further quantitative analysis. Automated quantification of the highly complex and rapidly changing brain morphology in MRI data could improve the diagnostic and decision-making processes.

Image segmentation is an early step for the volumetric quantification of the fetal brain. Shape or volume information could be relevant to the developing cortex, cerebellum, brainstem, white matter, and cerebrospinal fluid spaces [2,3]. The automatic segmentation of the developing human brain is a primary step for analysis, as manual segmentation is time-consuming and may be prone to human error. However, fetal brain segmentation based on SR fetal brain volumes is still challenging due to artifacts that are blurry or caused by motion, rapidly changing fetal brain anatomy, and the effects of partial volume.

Various atlas-based methods have been developed for brain tissue segmentation [4]. However, these methods need an atlas, which now only exists for normally developing fetuses. Falick et al. [5] used single-class high-resolution fetal brain volumes for fetal brain segmentation, but multiclass segmentation still needs to be explored. Deep-learning-based segmentation models have recently been employed to segment the fetal brain into different tissue types by using low-resolution coronal-direction slices to handle fetal brain tissue segmentation problems [6].

Faghihpirayesh et al. [7] used an encoder–decoder UNet model with multiple branches and skip connections to maintain high accuracy while devising a parallel combination of convolution and pooling operations. They used a private dataset to train their proposed model. However, they only handled the single-class segmentation problem by using 2D slices, which is not challenging and quite simple. A 2D segmentation model for volumetric 3D segmentation cannot handle temporal relationships, unlike 3D segmentation models. Moreover, they used only binary class segmentation, while the proposed model addresses the problem of multi-tissue fetal brain segmentation. Asis et al. [8] used an end-to-end generative adversarial neural network (GAN) to segment the fetal brain in functional magnetic resonance images (rs-fMRI). They segmented the full fetal brain and handled binary class problems by using a private dataset. Unlike the models in these works, the proposed multi-view segmentation model can handle the 3D segmentation of volumetric data by using a stacking approach to multi-view segmentations.

Zhao et al. [9] trained a patch-based 3D segmentation model for fetal brain segmentation by using an in-house dataset. This 3D segmentation model required powerful computational resources. However, the 3D-CNN holds great potential for fully utilizing the 3D information from MRI data, which also contain multi-view information. However, 3D-CNN-based segmentation greatly increases the network scale and computational cost [10]. It should be noted that the major bottleneck in the development of segmentation algorithms for medical imaging is the lack of data—either the availability of atlases for atlas-based segmentation or that of training data for supervised machine learning methods. In addition, there is still a need to explore and implement deep-learning-based approaches, as no clear benchmark is available for fetal brain segmentation.

In turn, the dating of the precise gestational age (GA) is essential for assessing pregnancy, fetal development, and neonatal care. Before sonography, obstetricians routinely relied on the last menstrual period for the dating of the gestational age in pre-birth life [5]. The crown-rump length (CRL) method is used in the first trimester to estimate gestational age. Other methods are used in the last two trimesters, such as brain bi-parietal diameter, head circumference, femur length, and abdominal circumference. These methods were reported decades ago and are still used today [5].

Though sonographic assessment during the first trimester is the most well known and accurate method for estimating the gestational age, it shows large variations in the second and third trimesters due to the variability in organ size. According to previous studies, the assessment of gestational age by combining the above-mentioned biometric data can achieve an accuracy of ±7 to 10 days for the second trimester and ±21 to 30 days for the third trimester. Various methods, such as the measurement of the cerebellar length and the transcerebellar diameter, accurately predict gestational age in singleton and twin pregnancies [11]; however, they require good visualization of the cerebellum by specialized sonographers.

In summary, estimations made with sonographic measurements are strongly affected by the inherent variability in organ size and the intrinsic signal properties of ultrasonography [12]. The inaccuracy of sonographic assessment has driven the need to find different approaches that can be used to accurately determine gestational age. MRI is gradually being recognized as a powerful helper for ultrasonography in the evaluation of the fetal brain. MRI-based methods provide a high resolution, soft-tissue contrast, and visibility of the whole brain independently of fetal presentation [13,14]. As pregnancies advance, the biological variations among normal fetuses increase, and the ranges of values of each biometric measurement associated with a specific GA also increase. This means that while the predictive error at ±10 days GA is considered acceptable in most clinical settings, the predictive error at ±18 days is estimated to offer little clinical value [15]. Therefore, when screening occurs in the second and third trimesters, the error margins produced by current methods are highly increased; thus, they are not clinically useful. Accordingly, there is a need to develop an alternative technique for estimating the GA.

Fung et al. [16] developed a machine learning (ML) model for estimating the GA and predicting future growth. They utilized multi-center, international, and population-based project data from the International Fetal and Newborn Growth Consortium for the 21st Century (INTERGROWTH-21st). Kojita et al. [17] developed VGG-based transfer learning models for GA prediction. They employed an in-house (private) dataset. The deep learning model was trained with T2-weighted images from 126 training cases and 29 validation cases. The remaining 29 cases were utilized as test data, with the fetal age being estimated by the model and by using BPD (biparietal diameter) measurements. They drew a relationship between the estimated and standard gestational ages by using Lin’s concordance correlation (ρc). The model’s outcome in terms of concordance was significant (ρc = 0.964).

Furthermore, Lu et al. [18] developed machine learning models that could provide accurate estimations for obstetricians alongside traditional clinical practices and an efficient and effective supporting tool for pregnant women for self-monitoring. A total of 4212 intrapartum recordings were selected, of which 3370 samples were used as the training set and 842 samples were used as the test set. In addition, several simple and powerful machine learning algorithms were trained, and their performance was evaluated with real test data. The experimental results showed an intersection over union (IoU) of 0.64 between the predicted range of fetal weight at any gestational age from the ensemble model and that from ultrasound. Using their private dataset, they used simple clinical features with traditional machine learning models for the prediction of gestational age and weight. No deep-learning-based models were used as a comparison with the machine learning models. No efficient feature engineering approaches were used to predict gestational age.

Alliance et al. [19] developed a novel method based on machine learning models and used each subset of predictors based on an ensemble model constructed by using the Super Learner algorithm. The resulting model was a weighted average of multivariate adaptive regression splines, random forests, gradient boosting, support vector machines, and multiple linear regression. They assessed the diagnostic accuracy by using the receiver operating curve (AUC) and Bland–Altman analysis. They collected datasets from population-based cohorts in five countries (Bangladesh, Ghana, Pakistan, Tanzania, and Zambia). Women at <20 weeks of gestation according to ultrasound-based dating were used as a study case for the prediction of gestational age. A total of 7428 liveborn infants were included. This dataset is not publicly available. The resulting model was a weighted average of multivariate adaptive regression splines, random forests, gradient boosting, support vector machines, and multiple linear regression. They achieved the highest AUC of 0.96. They used only clinical features with traditional machine learning models for the age prediction. No imaging-based features were used to evaluate the performance of the machine learning or deep learning models for the prediction of gestational age.

Payette et al. [20] employed deep learning models such as ResNet-18 and ResNet-50 with a combination of different layers for the prediction of gestational age. They collected 741 fetal brain MRIs in order to predict fetal gestational age (in days). The authors proposed a basic ResNet18-based regressor model that used a private dataset, and they did not use any other segmentation-based or 3D volumetric-based features for gestational age prediction. They used cropped 2D images covering the fetal area only in the input images to train the basic ResNet18 with overall global features, and this could efficiently help in the extraction of local image features for the prediction of fetal gestational age.

Shen et al. [21] used attention-guided, multi-plane ResNet-50 models trained on Stanford data to predict the gestational age. They trained various CNN models based on only imaging features for the prediction of gestational age. Imaging features might not be sufficient to accurately predict gestational age. However, we used various feature extraction approaches, including imaging, radiomics, 3D latent space autoencoder-based features, and deep features extracted from the last layer of multi-view 2D image slices from segmented brain tissues, to extract more localized features for the prediction of gestational age. The fusion of multi-scale segment-based deep features achieved better performance than that of the state-of-the-art methods.

There is a further need to investigate different methods with deep learning models for GA prediction. The existing methods are based on single-feature extraction techniques that use basic deep learning models. Correspondingly, the datasets used for the existing methods are in-house and private. There is a need to set a benchmark on a publicly available dataset for further comparisons and enhancements in deep learning/machine learning for the prediction of gestational age and segmentation of the fetal brain.

To solve the above-mentioned issues, we propose effective yet automatic methods for the segmentation of fetal brain tissue and prediction of gestational age. To the best of the authors’ knowledge, this is the first paper to propose an end-to-end solution for fetal brain segmentation in MRI images and GA prediction. Deep learning is the basis for the proposed fetal segmentation method, IRMMNET (inception residual multi-scale multi-view network). By effectively combining segmentation maps from the axial, coronal, and sagittal views to create a 3D segmentation volume, IRMMNET incorporates important insights from multi-view MRI. IRMMNET consists of several layers with the capacity of reusing features and information at several scales and depths. The inception residual encoder block (EB) and the dense spatial attention (DSAM) block are two proposed blocks that are part of IRMMNET. The EB aids in extracting information from multi-view MRI scans that is pertinent to multi-scale fetal brain tissue. The DSAM improves feature reuse while lowering the model’s parameter count. The EB and DSAM help segment small lesions that have a small number of semantic pixels that are missed by traditional encoder–decoder networks. Then, we propose three methods for GA prediction—GA prediction using the IRMMNET segmentation model’s encoder, GA prediction using a 3D autoencoder, and GA prediction using radiomics features. The implementation of the proposed method will be available at https://github.com/Moona-Mazher/Fetal-Segmentation-Gestational-Age-Prediction-Deep-Learning on 20 November 2022.

The following is a list of this paper’s major contributions:

1. Proposal of a novel multi-view multi-scale 3D fetal brain segmentation method named IRMMNET. It combines the key insights from multi-view MRI, including the axial, coronal, and sagittal views. IRMMNET comprises different layers with feature reuse capabilities and with various depths and multi-scale information. An efficient method for fusing segmentation maps of the axial, coronal, and sagittal views to develop a 3D segmentation volume is also presented.
2. Presentation of two effective blocks: the inception residual encoder block (EB) and the dense spatial attention (DSAM) block. The EB helps the fetal brain segmentation network extract multi-scale fetal-brain-tissue-relevant information from multi-view MRI images. The DSAM block enhances feature reuse and substantially reduces the number of parameters of the segmentation model. Extensive experiments were performed with various combinations and settings of the fetal brain segmentation model.
3. Proposal of three approaches to predicting GA: GA prediction by utilizing the IRMMNET segmentation model’s encoder, GA prediction by utilizing a 3D autoencoder, and GA prediction by utilizing radiomics features. The explainability and importance of the radiomics features are also presented.
4. Demonstration of the generalization capabilities of the proposed fetal brain segmentation and GA prediction methods on two different tasks: the segmentation of head and neck tumors and the prediction of patients’ survival days.

The rest of this paper is presented as follows: Section 2 introduces the proposed multi-view multi-scale 3D fetal brain segmentation method and the proposed fetal age prediction method. Section 3 presents the datasets used in our study, the proposed fetal brain segmentation model’s results, and the GA prediction method’s results. Section 3.5 discusses the generalization capabilities of the proposed methods. Section 4 discusses the findings of the study and its limitations. Section 5 concludes the paper and presents the future work.

## 2. Methodology

In this section, we explain the proposed multi-view multi-scale 3D fetal brain segmentation method (Section 2.1) and the proposed fetal age prediction method (Section 2.2).

### 2.1. Proposed Multi-View Multi-Scale 3D Fetal Brain Segmentation Method

**Figure 1:**
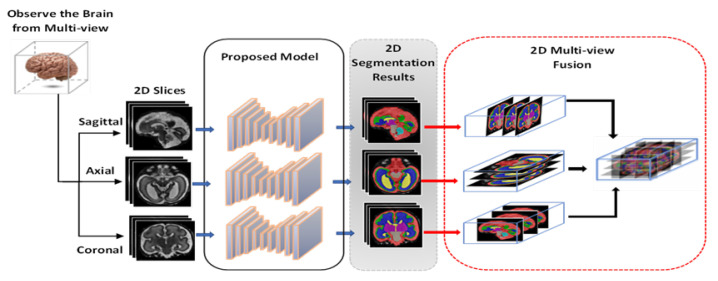
A schematic diagram of the proposed method for 3D fetal brain segmentation from the axial, coronal, and sagittal views.

Figure 1 presents a schematic diagram of the proposed multi-view multi-scale 3D fetal brain segmentation method. The three available views—axial, coronal, and sagittal—are inputted into the proposed segmentation model to generate a 2D segmentation mask for each view. Then, the resulting segmentation masks are combined to construct a 3D segmentation map. Specifically, we stack a 2D segmentation mask of each view to form a predicted 3D segmentation mask. This process can be expressed as follows:

(1) $$\begin{array}{c} O_{A}=\mathrm{orgmax}(\mathrm{Sigmoid}(L_{A})),\\ O_{S}=\mathrm{orgmax}(\mathrm{Sigmoid}(L_{S})),\\ O_{C}=\mathrm{orgmax}(\mathrm{Sigmoid}(L_{C})) \end{array}$$

(2) $$\begin{array}{cc} \; & 3DV\left[:,:,A\right]=\sum_{i=1}^{N}O_{A},\\ \; & 3DV\left[:,S,:\right]=\sum_{i=1}^{N}O_{S},\\ \; & 3DV\left[C,:,:\right]=\sum_{i=1}^{N}O_{C} \end{array}$$

where $L_{A}$ represents linear-layer neurons using the axial view, $L_{S}$ using the sagittal view, and $L_{C}$ using the coronal axis view.

(3) 3DV[:,:,:]=3DV[:,:,A]+3DV[:,S,:]+3DV[C,:,:]

where $O_{A}$ stands for the output prediction of the proposed model when using axial slices, $O_{S}$ is the prediction of the proposed model when using the sagittal view, and $O_{C}$ is the output when using coronal slices; 3DV is the 3D prediction volume that is reconstructed by stacking up the predicted 2D slices from each 2D view ($O_{A}$, $O_{s}$, and $O_{c}$).

#### 2.1.1. Multi-View Multi-Scale Segmentation Network

**Figure 2:**
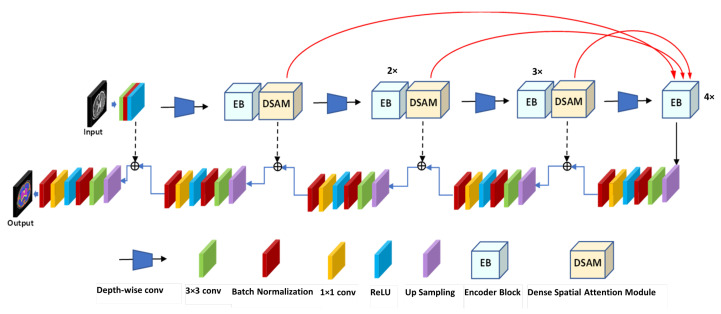
The proposed IRMMNET multi-view multi-scale segmentation network.

Figure 2 presents the proposed multi-view multi-scale segmentation network. The proposed model is designed based on the concept of an encoder–decoder with skip connections. The proposed inception residual encoder block (EB) and dense spatial attention (DSAM) block are used in the encoding path. In contrast, an efficient yet simple 2D convolutional layer module with 2D upsampling layers, including regularization layers, is used on the decoding path.

In the encoding path, a DSAM block is used in each encoder block, which sends information at every block from each encoder layer to the bottom layer. The number of channels is doubled at each EB block, and the input size of the feature maps is reduced by half based on the depth-wise convolutional layer in the encoding path. An increasing number of feature map blocks are used in each stage of encoder blocks; the number of EB blocks progressively increases at each stage of the encoder side. The first encoder block uses one EB block. Similarly, the second, third, and fourth blocks employ 2, 3, and 4 inception residual blocks, respectively.

The red lines in Figure 2 highlight the multi-scale feature maps from each encoder block fed to the model’s bottom layer. This strategy increases the efficiency of the feature maps by reusing and fusing the feature information at the level of downsampling. In the proposed segmentation model, the features are extracted from three levels (red lines), which enables the model benefit from the multi-scale transformation of high-level semantic information and low-level information of the position and texture. Three downsampling layers that carry features’ information are passed to the bottom-layer module to guarantee an improved cross-level feature connection and complementarity in cross-level information.

In the decoding path, the size of the feature maps increases after each 2D upsampling layer, and the original size of the training input images returns in the output in the final layer. The first 2D upsampling layer comprises two efficient convolutional layers (2D 1 × 1 Conv, 2D 3 × 3 Conv) with a BN and ReLU layer. To reconstruct the semantic information, the feature maps are concatenated with each encoder and decoder block. The BN and ReLU regularization layers are used with 2D upsampled layers and 2D 3 × 3 Conv layers for smooth optimization and training of the proposed model. The 1 × 1 Conv layer and sigmoid activation function are used for the reconstruction of the segmentation map.

Below, we explain the architectures of the proposed EB and DSAM blocks.

#### 2.1.2. Inception Residual Encoder Block (EB)

**Figure 3:**
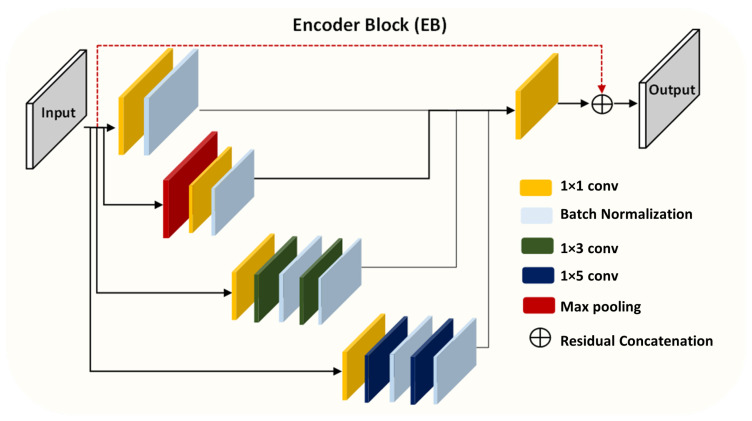
The schematic diagram of the proposed inception residual encoder block (EB).

Figure 3 depicts the proposed inception residual encoder block. Unlike in the Inception-Res architecture [22], we introduce a batch normalization (BN) layer after each convolutional layer, except for the bottleneck layers. In addition, we use 1×1 and 1×3 kernels with a 1×5 kernel branch, which was inspired by the DeepLab architecture [23].

It should be noted that the batch normalization layer produces smooth training and can avoid gradient vanishing while retaining the convolutional layers. The feature maps are aggregated by convolving them with three kernels: 1×1, 1×3, and 1×5. It is assumed that $x_{l}$ is the input and $x_{l+i}$ is the output of the ith layer. $c_{1\times n}$ is a 1×n kernel convolutional layer and $c_{b}$ represents the batch normalization layer. $c_{1\times 1}$ denotes the bottleneck layer. The output of each EB module from the encoder path can be expressed as follows:

(4) $$\begin{array}{c} x_{l+1}=l_{1}\times l_{2}\times l_{3}\times l_{4} \end{array}$$

(5) $$\begin{array}{c} l_{1}=\left(c_{1\times 1}\left(k\right)\right)c_{b} \end{array}$$

(6) $$\begin{array}{c} l_{2}=Maxpool\left(c_{1\times 1}\left(k\right)c_{b}\right) \end{array}$$

(7) $$\begin{array}{c} l_{3}=c_{b}\left(c_{1\times 3}\left(c_{b}\left(c_{1\times 3}\left(c_{1\times 1}\left(k\right)\right)\right)\right)\right) \end{array}$$

(8) $$\begin{array}{c} l_{4}=c_{b}\left(c_{1\times 5}\left(c_{b}\left(c_{1\times 5}\left(c_{1\times 1}\left(k\right)\right)\right)\right)\right) \end{array}$$

where $k=x_{l}$.

#### 2.1.3. The Proposed Dense Spatial Attention Module (DSAM)

**Figure 4:**
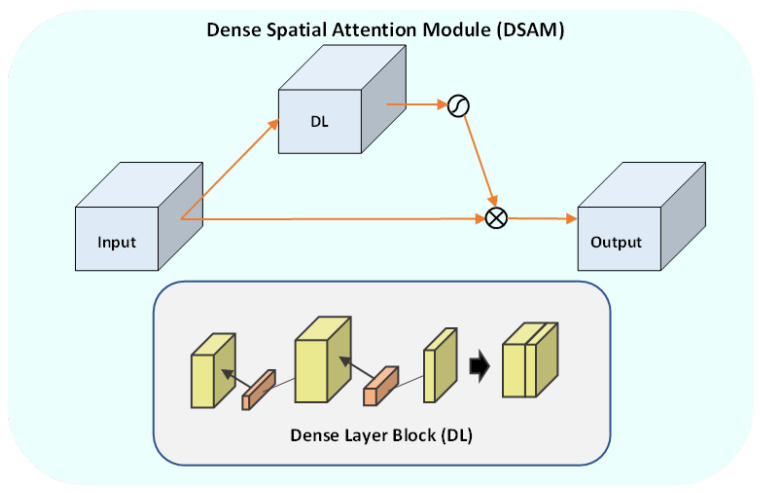
A schematic diagram of the proposed dense spatial attention module (DSAM). DL is a basic dense layer. The input x is multiplied by the DL and by the swish activation function (x∗DL∗(x/(1+e−x))).

It should be noted that the attention modules that are often used in image segmentation and object detection models are mainly divided into channel-wise attention and point-wise attention modules, and the representatives of these two attention models are squeeze and excitation (SE) and the spatial attention module (SAM) [24]. A channel attention map exploits features’ inter-channel relationships, and the feature map obtained from the channel attention is considered a feature detector. The spatial attention module focuses on what is meaningful given an input image through the benefit of the combination of average pooling and max pooling. Figure 4 shows a schematic diagram of the DSAM. As shown, we modified the SAM by replacing the point-wise convolutional layer with a basic dense layer (DL). We also used the swish activation function instead of the sigmoid function [25], as swish is less prone to vanishing gradient problems. The swish activation function can be expressed as follows:

(9) Swish(x)=x.sigmoid(x)

where *x* is the input feature map.

In the DL, the feature maps of all preceding layers are used as inputs, and their feature maps are used as inputs for all subsequent layers. The output of each dense block is concatenated with every previous dense block. The operation of the DL can be expressed as follows:

(10) $$DL=[x,B_{1}\left(x\right),B_{2}\left(\left[x,B_{1}\left(x\right)\right]\right),B_{3}\left(x,B_{1}\left(x\right),B_{2}\left(\left[x,B_{1}\left(x\right)\right]\right)\right),\ldots ]$$

where $B_{1}\left(x\right)$ is dense block 1, $B_{2}$ is dense block 2, and so on. *x* is the input feature map. We used 12 dense blocks in our case. The output of the proposed DSAM block, $DSAM_{out}$, can be formulated as follows:

(11) $$DSAM_{out}=x*DL*\left(x/\left(1+e^{-x}\right)\right)$$

The key advantages of the DL are (1) the alleviation of the vanishing gradient problem, (2) the strengthening of feature propagation, (3) the encouragement of feature reuse, and (4) the substantial reduction in the number of parameters. The feature maps are concatenated from the previous layer to the next layer to build the dense block in the proposed dense layer. The feature maps keep the relevant information from every layer and are reused in the final layer to get semantic information. The proposed DSAM uses the dense block to provide better semantic information and improves the flow of gradient information for easy training of the proposed model. The DSAM block reduces the problem of overfitting with smaller training set sizes by using dense connections. DSAM blocks also provide direct access to the gradients from the loss function and the original input signal, which leads to implicit deep supervision. It is worth noting that all layer weights in the proposed model were trained from scratch with the FeTA dataset.

#### 2.1.4. Loss Function and Implementation Details

In this paper, we employ the Combo loss function [26] to train the proposed model with multi-class settings for fetal brain tissue segmentation. Combo loss can be expressed as follows:

(12) $$\begin{array}{c} L=\alpha \left(-\frac{1}{N}\sum_{i=1}^{N}\beta \left(t_{i}-\ln p_{i}\right)+\left(1-\beta \right)\left[\left(1-t_{i}\right)\ln\left(1-p_{i}\right)\right]\right)-(1\\ -\alpha )\sum_{i=1}^{K}\left(\frac{2\sum_{i=1}^{N}p_{i}t_{i}+S}{\sum_{i=1}^{N}p_{i}+\sum_{i=1}^{N}t_{i}+S}\right) \end{array}$$

where $t_{i}$ is the one-hot-encoded target or ground truth, $p_{i}$ is the predicted probability, *N* is the number of classes multiplied by the number of samples, and the *S* is a small constant number that is added to prevent division by zero. α controls the amount contributed by the Dice term in the loss function *L*. β∈[0, 1] controls the level of penalization of the model for false positives/negatives. The *S* term is added to prevent division by zero. The *S* constant is added in both the denominator and the numerator of the Dice term.

All models were trained using an Adam optimizer with a learning rate of 0.0001, ρ = 0.95, ϵ = $1*10^{-8}$, and decay = 0. Based on the experimental evaluations, it was found that α = 0.5 for the Dice and cross-entropy terms produced the best results. Different values of β were tried for all datasets, and it was found that β = 0.5 was the best value for the proposed dataset for the segmentation task.

### 2.2. Approaches to the Prediction of Gestational Age (GA)

This subsection proposes three approaches to predicting GA: (1) GA prediction by utilizing the IRMMNET segmentation model’s encoder, (2) GA prediction by utilizing a 3D autoencoder, and (3) GA prediction by utilizing radiomics features.

#### 2.2.1. GA Prediction by Utilizing the IRMMNET Segmentation Model’s Encoder

**Figure 5:**
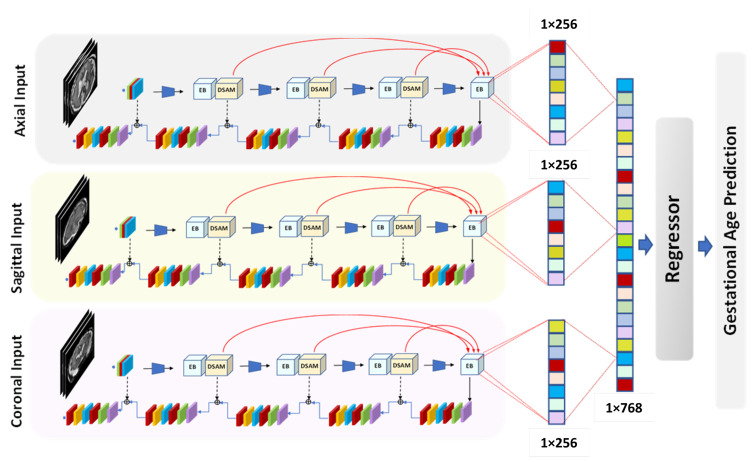
GA prediction by utilizing the IRMMNET segmentation model’s encoder.

The encoder of the proposed IRMMNET segmentation model can automatically learn multiple filters in parallel and extract low- and high-level features, such as edges, intensities, and textures. Different filters capture various characteristics of the input images that are used in GA prediction. Figure 5 presents the proposed framework for GA prediction by utilizing the IRMMNET segmentation model’s encoder. As one can see, deep features are extracted from the trained encoder of each view’s trained segmentation model. Each view’s encoder generates a feature vector with dimensions of 1×256. It should be noted that the feature vectors of all slices of each volume are combined to produce one feature vector that represents the whole volume. The feature vectors of the three views are concatenated to form one single feature vector with dimensions of 1×768. Different regression algorithms are trained with the extracted feature vector in order to predict the GA.

#### 2.2.2. GA Prediction by Utilizing a 3D Autoencoder

**Figure 6:**
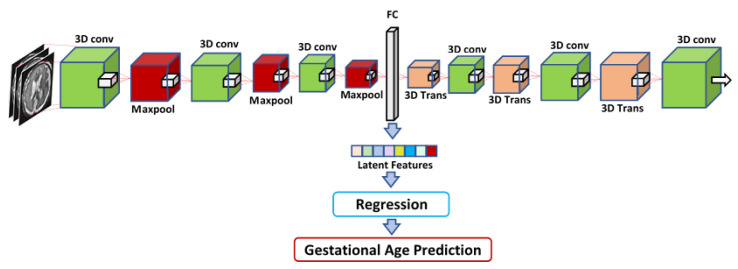
GA prediction by utilizing a 3D autoencoder. The latent features are extracted by using input image volumes for GA prediction.

Figure 6 presents the proposed approach to GA prediction by utilizing a 3D autoencoder. The 3D autoencoder consists of an encoder- and decoder-based model. A 3D volume is fed to the proposed 3D autoencoder to extract latent space features. As shown in Figure 6, the encoder part of the 3D autoencoder consists of a 3D convolutional layer, batch normalization layer, ReLU layer, and 3D maxpool layer, and the decoder side of the 3D autoencoder comprises a 3D transposed layer at each level on that side along, with a 3D convolutional layer and a batch normalization layer.

On the encoder side, the volume is reduced at each encoder block, while the volume is increased at each decoder block. The 3D transposed layer is used to upsample the feature maps at each decoder block. The bottom layer represents the 3D volume in the lower dimension. After flattening the lower dimension, a latent vector is produced. Different regression algorithms are trained with the latent features to predict the GA, as shown in Figure 6.

#### 2.2.3. GA Prediction by Utilizing Radiomics Features

**Figure 7:**
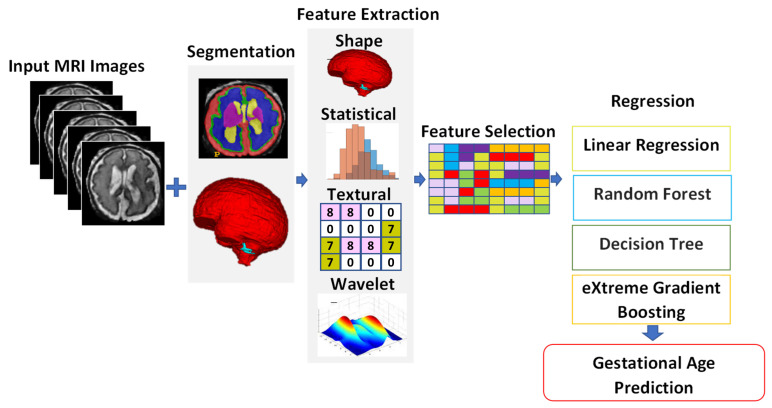
GA prediction by utilizing radiomics features.

Figure 7 shows the proposed approach to GA prediction by utilizing radiomics features. Here, we also extract 2D slices of the axial, sagittal, and coronal views from a 3D volume dataset. We train the proposed segmentation model for the dataset containing the three views and stack the output of each 2D view to reconstruct a 3D volume. The radiomics features are extracted from the input volumes of the dataset. The radiomics features used include shape-based, statistical, and wavelet features. Among the 108 radiomics features, a set was chosen based on a correlation-based feature selection technique [27]. The selected radiomics features were elongation, flatness, major axis length, minor axis length, max 3D diameter, sphericity, surface area, energy, entropy, kurtosis, mean, skewness, coarseness, contrast, correlation, inverse-diff moment, complexity, and strength. The radiomic features were extracted from Feta MRI images by using the Pyradiomics package [28]. Different regression algorithms were trained with the extracted radiomics features to predict the GA.

#### 2.2.4. Regression Techniques for GA Prediction

Different regression techniques were tested on the features extracted with the three approaches mentioned above for the prediction of the GA. We found that four regression techniques give acceptable results: random forest (RF) [29], regression trees (RT), linear regression (LR), and extreme gradient boosting (XGB) [30]. RF is supervised by traditional machine learning and is widely used for classification and regression problems. It uses a bootstrapped dataset as a subset, picks random subsets of features, and runs random trees in parallel while building the trees. We set the number of trees to be from 100 to 1000 to create the forest. LR is a linear model that builds a linear relationship between input variables and a single output variable. RT is a tree-based regression model that trains a model by observing the input object’s features and generating a continuous output. The gradient-boosting regressor is a method that uses an additive forward model and allows arbitrary differentiable loss functions for optimization during training. The gradient-boosting model is an ensemble model that can be used for regression, classification, and predictive modeling problems. Extreme gradient boosting (XGB) is an open-source approach to gradient-boosting regression.

## 3. Experimental Results and Discussion

In this section, we explain the dataset used in our study (Section 3.1), present and discuss the results of the proposed fetal brain segmentation model (Section 3.3), and analyze the results of the GA prediction models (Section 3.4).

### 3.1. Dataset Description

The dataset included 80 T2-weighted fetal brain reconstructions with a corresponding label map that was manually segmented into seven different tissues/labels [20]. The seven labels were external cerebrospinal fluid (ECF), fluid gray matter (FGM), white matter (WM), ventricles (VCs), cerebellum (CBM), deep gray matter (DGM), and brainstem (BSTM). The dataset consisted of clinically acquired fetal brain reconstructions of both neurotypical and pathological brains with a range of gestational ages. The data were acquired using 1.5T and 3T clinical GE whole-body scanners (Signa Discovery MR450 and MR750) with either an eight-channel cardiac coil or a body coil. T2-weighted single-shot fast spin echo sequences were acquired with an in-plane resolution of 0.5mm×0.5mm and a slice thickness of 3 to 5 mm. The sequence parameters were the following: TR: 2000–3500 ms; TE: 120 ms (minimum); flip angle: $90^{\circ }$; sampling percentages: 55%.

**Figure 8:**
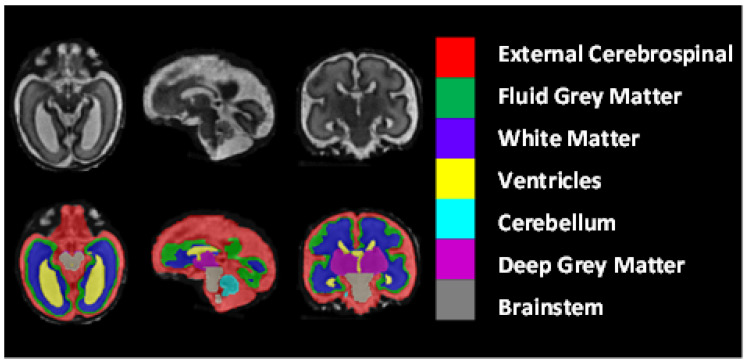
The class-mapping function for the axial, sagittal, and coronal slices.

Figure 8 shows the class-mapping function for the axial, sagittal, and coronal slices. The different colors show the seven classes used to predict fetal tissue segmentation.

Two different methods were used to create a super-resolution reconstruction of the fetal brain for each case from the acquired low-resolution axial, coronal, and sagittal images. Equal numbers of cases were used in the training and the evaluation datasets for each reconstruction method. For each case, the gestational age in weeks and the neurotypical/pathological label was given, in addition to the label maps.

The dataset was divided into 80% for training and 20% for testing. There were totals of 64 subjects in training and 16 subjects in the testing phase.

### 3.2. Evaluation Metrics

In this study, four metrics were used to assess the performance of the fetal brain segmentation models: Dice (DSC), Hausdorff distance (HD95), sensitivity, and specificity, which are are commonly used for the validation of medical volume segmentation approaches [31]. This is also called the overlap index. It measures the overlap between ground-truth (GT) and predicted segmentation masks. For the GT and predicted masks, DSC is defined as follows:

(13) Dice(X,Y)=2|X∩Y|/|X∪Y|

Hausdorff distance (HD95): The HD95 is calculated as the mean of two directed 95% Hausdorff distances:

(14) $$HD95=\frac{\vec{d}H,95\left(X,Y\right)+\vec{d}H,95\left(Y,X\right)}{2}$$

where X is the ground truth (GT) and Y is the predicted mask. HD is the maximum distance between the sets of points X and Y and between Y and X.

Sensitivity is used to compute the positive portion of voxels by using the ground-truth (GT) and predicted segmentation masks.

(15) Sensitivity=TRP=TP/(TP+FN)

where TRP is the true positive rate, TP is true positive, and FN is false negative.

Specificity is also called the true negative rate (TNR), and it is used to compute performance based on the GT and predicted segmentation masks.

(16) Specificity=TNR=TN/(TN+FP)

where TP is true positive, FP is false positive, TN is true negative, and FN is false negative.

In addition, we use the root-mean-square error (RMSE) and concordance (C-index) to evaluate the GA prediction models. The C-index is used to compute the correlation between the predicted gestational age and ground-truth gestational age. The RMSE can be expressed as follows:

(17) $$RMSE=\sqrt{\frac{\sum {\left(GA_{pre}-GA_{GT}\right)}^{2}}{N}}$$

The C-index can be formulated as follows:

(18) $$C\text{-}\mathrm{index}=concordance-index(GA_{GT},GA_{pre})$$

where $GA_{pre}$ is the predicted value, $GA_{GT}$ is the ground-truth value for the *i*th observation in the dataset, and N is the sample size.

### 3.3. Performance Analysis of the Proposed Segmentation Model

#### 3.3.1. Ablation Study

Table 1 tabulates the DSC, HD95, sensitivity, and specificity values of the proposed segmentation model, IRMMNET, with the axial, sagittal, and coronal views (IRMMNET-Axial, IRMMNET-Sagittal, and IRMMNET-Coronal). IRMMNET-Coronal obtained better performance for all classes than IRMMNET with the axial and sagittal planes. It achieved DSC, HD95, sensitivity, and specificity scores of 0.789, 21.56, 0.818, and 0.976, respectively.

**Figure 9:**
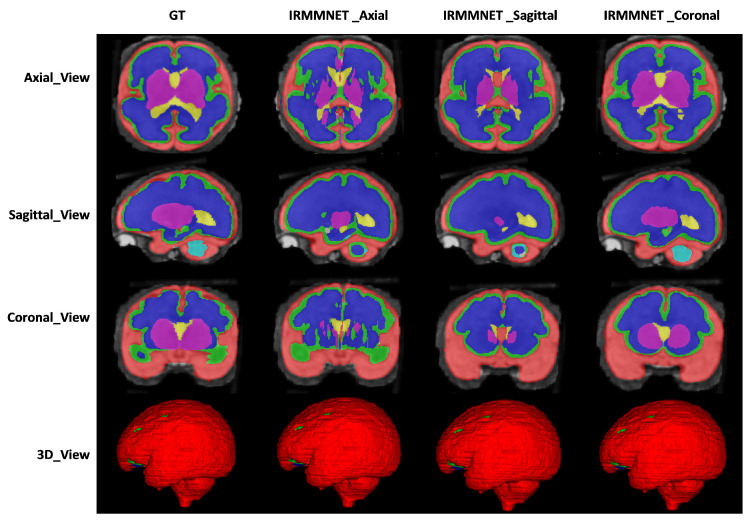
The segmentation maps of the proposed model with different views.

Figure 9 shows the proposed IRMMNET model’s segmentation maps for the axial, sagittal, and coronal planes. In addition, 2D and 3D volumetric views of the segmentation images are shown in Figure 9 for the axial, sagittal, and coronal planes. It can be visibly noticed that the coronal-view model generated accurate segmentation masks, in which the predictions for the deeper and smaller classes were close to the GT. It should be noted that the proposed model was initially tried on the 2D axial slices, but the predicted segmented images yielded a bad prediction for the deep classes. Therefore, the proposed segmentation model was applied to the three views’ 2D slices (axial, sagittal, and coronal) for fetal brain segmentation. Among the predicted segmentation results for the fetal brain, the 2D coronal view produced the best results.

In Table 2, we compare the proposed model with different segmentation models. Specifically, the basic UNet (BaseUNet) for 2D brain tissue segmentation was trained by using the axial, coronal, and sagittal views (BaseUNet-Axial, BaseUNet-Sagittal, and BaseUNet-Coronal). As one can see, BaseUNet-Coronal yielded the highest DSC score (0.728) and the lowest HD95 score (29.042). In addition, different ResUnet models were trained by using the axial, coronal, and sagittal views (ResUnet-Axial, ResUnet-Sagittal, and ResUnet-Coronal) for 2D brain tissue segmentation. In the ResUnet model, the residual blocks were added to the base UNet model. The results of the three ResUnet models were better than those of the BaseUNet models. Finally, we added squeeze-and-excitation (SE) blocks into ResUNET (SE-ResUNet) and trained it on the three views, yielding SE-ResUNet-Axial, SE-ResUNet-Sagittal, and SE-ResUNet-Coronal. However, SE-ResUNet-Coronal had an improved DSC score compared to that of ResUnet-Coronal; its HD95 and specificity values were worse. As shown in Table 2, the proposed IRMMNET model comparatively produced a better performance with the axial and sagittal views. However, IRMMNET-Coronal achieved a DSC score of 0.789 and HD95 score of 21.565, which were better than those of all models used for comparison.

To enhance the proposed model’s prediction, we fused the axial, sagittal, and coronal outputs of the proposed model to create a so-called multi-view model, which provided a 3D segmentation map. Later, the performance was evaluated by using the predicted 3D segmentation map achieved with our three multi-view models and a GT segmentation map. It resulted in a better estimation in terms of the Dice, HD95, sensitivity, and specificity scores. We constructed a 3D segmentation map from the three views of the proposed model and evaluated the performance by using the predicted 3D segmentation map (achieved with our three multi-view models) and ground-truth segmentation map. Table 3 presents an ablation study of the proposed IRMMNET. Although the baseline Multi-view-2D-Inception+Residual model achieved optimal performance, its performance was upgraded when the DSAM module (i.e., Multi-view-2D Inception + Residual + DSAM) was systematically added. However, adding the multi-scale feature approach to the Multi-view-2D-Inception+Residual+DSAM model with the fusion of multiple views (i.e., IRMMNET) produced the highest performance scores in comparison with those of all of the state-of-art-methods and the baseline model for the fetal brain segmentation task when using the FeTA 2021 dataset. IRMMNET achieved DSC, HD95, sensitivity, and specificity scores of 0.791, 21.66, 0.819, and 0.980, respectively.

We also studied the efficacy of different loss functions with the proposed model. As tabulated in Table 4, the proposed Combo loss function achieved better performance than that of the binary cross-entropy (BCE) and Dice loss functions. No big improvements were noticed when we combined the Dice loss and BCE loss. However, the Dice loss produced the lowest scores.

The training and validation times were also estimated for the proposed and the state-of-the-art methods for a comparison of the computational costs. The training time of our proposed solution was 55 min, and the time taken for validation was less than 2 min. The computational times for training and validation are given in Table 5.

We applied a Mann–Whitney U test or Wilcoxon Rank Sum test to compute the *p*-values between the predicted masks and their corresponding ground truths [32,33]. In segmentation tasks, the *p*-value needs to be higher than 0.05 to be statistically significant, unlike in classification tasks. A comparison of the statistical analyses of the proposed IRMMNET and the state-of-the-art methods is given in Table 6. In the table, a *p*-value that is greater than 0.05 represents a greater similarity between the predicted and ground-truth segmentation maps. Similarly, a higher *p*-value also represents an accurate segmentation, and vice-versa. The table shows that the proposed IRMMNET had consistent results, and it statistically validates the segmentation results.

**Figure 10:**
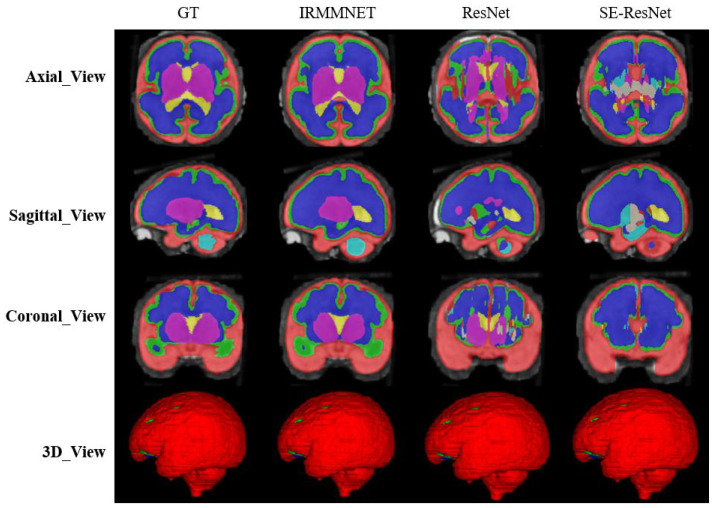
The segmentation results for the proposed Multi-view-IRMMNET, Multi-view-ResUNET, and Multi-view-SE-ResUNET models.

To validate the results of the proposed fused model (i.e., Multi-view-IRMMNET), we also applied the same fusion technique to the ResUNet and SE-ResUNet models, which yielded Multi-view-ResUNET and Multi-view-SE-ResUNET. The predicted 2D slices and 3D volumes of the proposed model, ResUnet, and SE-ResUNet are shown in Figure 10. Although ResUNet and SE-ResUNet successfully predicted outer classes, such as external cerebrospinal fluid (ECF), they failed to predict inner/deeper classes, like deep gray matter (DGM). It is conspicuous that the proposed model’s predictions for all classes were close to the given GT.

**Figure 11:**
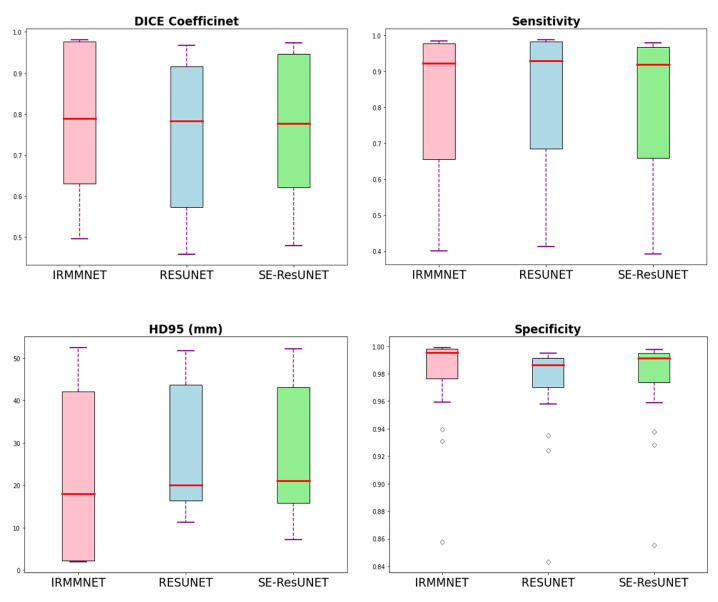
Box plots of the DSC, HD95, sensitivity, and specificity scores of the proposed Multi-view-IRMMNET, Multi-view-ResUNET, and Multi-view-SE-ResUNET models.

Table 7 presents the Dice, HD95, sensitivity, and specificity scores of the proposed Multi-view-IRMMNET, Multi-view-ResUNET, and Multi-view-SE-ResUNET models. The proposed Multi-view-IRMMNET model achieved the best segmentation results with a DSC of 0.791, HD95 of 21.66, sensitivity of 0.819, and Specificity of 0.980. The Multi-view-ResUNET model achieved less performance scores than those of Multi-view-IRMMNET. This model achieved a DSC of 0.758, and Multi-view-SE-ResUNET achieved a maximum DSC of 0.772. We can conclude that our proposed Multi-view-IRMMNET achieved the highest DSC score in comparison with the other models, namely, Multi-view-ResUNET and Multi-view-SE-ResUNET. Similarly, Multi-view-IRMMNET achieved the lowest HD95 score of 21.66, as compared to those of Multi-view-ResUNET and Multi-view-SE-ResUNET. Figure 11 shows the box plots of the DSC, HD95, sensitivity, and specificity scores of the proposed Multi-view-IRMMNET, Multi-view-ResUNET, and Multi-view-SE-ResUNET models. Multi-view-IRMMNET showed the highest Q3 quartile in the DSC and specificity plots. In contrast, Multi-view-IRMMNET’s median and maximum values remained higher in all plots compared to those of the Multi-view-ResUNET and Multi-view-SE-ResUNET models.

**Figure 12:**
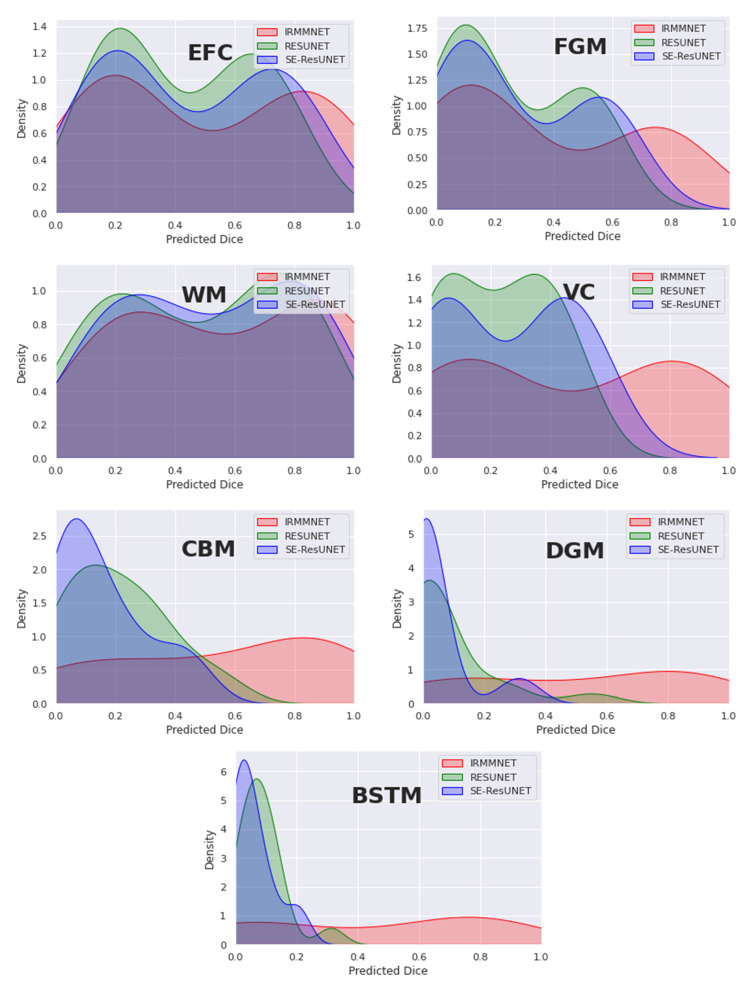
Density plots of the predicted 2D DSC for the proposed Multi-view-IRMMNET, Multi-view-ResUNET, and Multi-view-SE-ResUNET models.

Figure 12 overlays the density plots of the predicted 2D DSCs of the proposed Multi-view-IRMMNET, Multi-view-ResUNET, and Multi-view-SE-ResUNET models for each class. First, the distribution of the predicted 2D DSCs for Multi-view-IRMMNET was always significantly different from those of the Multi-view-ResUNET and Multi-view-SE-ResUNET models for all classes. This was especially the case for VC, CBM, DGM, and BSTM, which were the classes opposite to EFC, FGM, and WM. It is worth noting that when a class was absent in a segmented slice, the predicted 2D DSC was zero for that slice. This showed a small trend of zero appearing for all classes, especially for deep classes, such as VC, CBM, DGM, and BSTM.

Similarly, the distribution scores of all classes with Multi-view-IRMMNET were greater than those with Multi-view-ResUNET and Multi-view-SE-ResUNET. However, regardless of the class, the 2D DSC distributions of the Multi-view-ResUNET and Multi-view-SE-ResUNET models were always significantly different, were more shifted to the left, and had a larger standard deviation compared to that of the distribution of the proposed solution. The proposed solution was always shifted toward the higher values on the right.

#### 3.3.2. Comparing the Proposed Segmentation Model with Existing Methods

There has not been much research on the FeTA dataset and fetal brain segmentation, as private datasets are most commonly used in research. Table 8 compares the performance of the proposed Multi-view-IRMMNET with that of two state-of-the-art methods called DA_FaBiAN_Baseline [34] and TopoCP (2D) [35] in terms of DSC scores. As shown, Multi-view-IRMMNET achieved the best results, with a DSC score of 0.791. However, DA_FaBiAN_Baseline and TopoCP (2D) produced the same DSC score (0.70), but TopoCP (2D) obtained the smallest standard deviation.

### 3.4. GA Prediction Results

#### 3.4.1. Analyzing the Performance of GA Prediction Models

As mentioned in Section 2.2, three different methods were used to extract features from the MRI images of the fetal brain, and then these features were fed into a regression algorithm to predict the GA. Four different regression techniques were used for the regression of the input features: LR, XGB, RF, and RT. The first feature set, which was called IRMMNET Deepfeat, was extracted from the last encoder layer of the proposed IRMMNET model, as depicted in Figure 5. The second feature set, the so-called 3D deep autoencoder features, was extracted from the autoencoder depicted in Figure 6. The third feature set included radiomics features (Figure 7).

Table 9 shows that the RF regressor produced the lowest RMSE values with the radiomics, IRMMNET Deepfeat, and 3D deep autoencoder features. The RF regressor achieved the best GA prediction results with the radiomics features, with an RMSE score of 1.42 and a C-index score of 0.888. These results indicate that the proposed radiomics features used with the RF regressor were the most reliable method for predicting GA.

**Figure 13:**
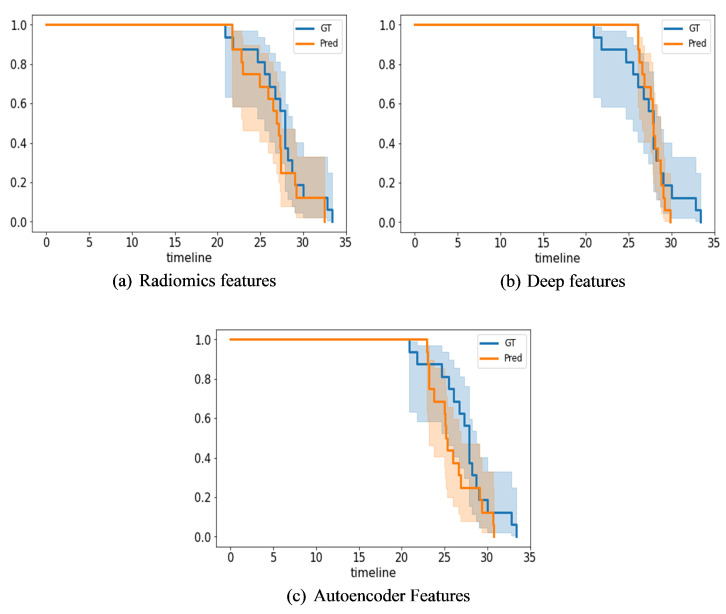
Kaplan–Meier plots of the radiomics, IRMMNET Deepfeat, and 3D deep autoencoder features with RF.

Figure 13 presents Kaplan–Meier plots of the radiomics, IRMMNET Deepfeat, and 3D deep autoencoder features. These plots show the gestational days predicted by the proposed GA prediction model in comparison with the ground truth for the validation datasets. Figure 13a shows the days predicted with the radiomics features with RF, Figure 13b shows the days predicted with the IRMMNET Deepfeat with RF, and Figure 13c shows the days predicted with the 3D deep autoencoder features with RF. As shown, the curves of the predicted days were very close to the GT curve for the radiomics-based features, unlike the curves for the IRMMNET Deepfeat and 3D deep autoencoder features. The curve of the predicted days that was produced based on the 3D deep autoencoder features was far from the curve based on the GT days. Hence, these curves prove the efficacy of the proposed GA prediction method based on radiomics features and RF.

#### 3.4.2. Analyzing the Explainability of the Radiomics Features

**Figure 14:**
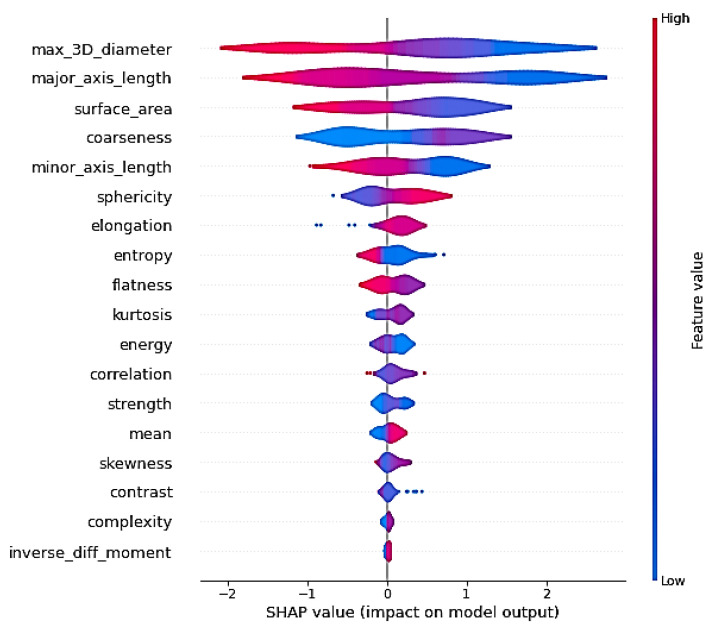
Feature importance of the radiomics features for GA prediction. The colors from red to blue represent the significance of the features in model prediction.

As shown above, the radiomics features yielded the best GA prediction results. Here, we employ the SHAP explainability method (SHapley Additive exPlanations) in order to analyze the most explainable radiomics features and their importance. SHAP is a game-theoretic approach to explaining the output of any machine learning model [36]. Figure 14 shows the feature importance of the radiomics features for the further analysis of the approach to using the radiomics features for GA prediction. We used the 18 best-explainable radiomics features with RF and obtained better scores than with all 108 extracted radiomics features. It was shown that the max 3D diameter and major axis length had the highest feature importance. We can say that the max 3D diameter and minor axis length were the most important features for GA prediction in comparison with the other features, which can help in future planning.

### 3.5. Generalization Capabilities of the Proposed Fetal Brain Segmentation and GA Prediction Models

To demonstrate the generalization capabilities of the proposed fetal brain segmentation model, we tested it on the Hecktor 2021 dataset [37]. A total of 224 training samples were provided. This dataset also contained clinical values and imaging samples. The dataset was divided into 80% for training and 20% for testing. The Hecktor 2021 dataset was converted into axial, coronal, and sagittal views. We trained the proposed IRMMNET fetal brain segmentation model with the axial, coronal, and sagittal views from a head and neck tumor dataset. Each volume in the Hecktor 2021 dataset had a spatial resolution of 144×144×144. The segmentation masks of the IRMMNET model for each view were fused to construct the 3D segmentation of the tumors in the head and neck dataset. Table 10 compares the results of the proposed model with those of existing state-of-the-art methods in terms of the DSC and HD95 scores. IRMMNET achieved a DSC score of 0.77 and an HD95 score of 3.02, which were better than those of the methods used for the comparison.

In addition, the proposed GA prediction method was tested on the the Hecktor 2021 dataset for the prediction of the patients’ survival days. The progression-free survival outcomes for all patients were provided in the CSV files with their clinical data and with various clinical variables. The head and neck tumor progression was based on the RECIST criteria: either an increased size of a known tumor (change in N and/or T) or the existence of a new tumor (change in M and/or N). Death due to a specific disease was also considered the progression of a disease that was previously considered stable.

Clinical variables, such as the patient age, patient gender, center ID, TNM group, M-stage, N-stage, T-stage, TNM edition, and chemotherapy status, were given with different values, and some variables had missing values. We used imputation to complete the missing values for all clinical features. We mapped integer values to each the individual N-, M-, and T- staging datum as follows: T-stage (Tx: 0, T1: 1, T2: 2, T3: 3, T4: 4, T4a: 5, T4b: 6), N-stage (N0: 0, N1: 1, N2:N2a: 3, N2b: 4, N2c: 5, N3: 6), and M-stage (Mx: 0, M0: 0, M1:1). In addition, the TNM group was also mapped to an ordinal categorical variable, which was based on the corresponding TNM stage information (7 I: 0, 8 I: 0, 7 II: 1, 8 II: 1, 7 III: 2, 8 III: 2, 7 IV: 3, 8 IV: 3, 7 IVA: 4, 8 IVA: 4, 7 IVB: 5, 8 IVB: 5, 7 IVC: 6, 8 IVC: 6).

The min/max normalization method provided by the scikit-learn Python package was used to normalize the clinical features’ values. A scaler was instantiated by using only the training data and then applied to the test set.

In these experiments, the RF regression technique was employed. As tabulated in Table 11, the clinical features obtained a C-index of 0.692. However, the C-index of the clinical features was lower than those of the radiomics, DeepFeat (deep features extracted from the encoder of the proposed IRMMNET model), and 3D deep autoencoder features, but it was better than those in existing studies, such as [39,40]. The Clinical + DeepFeat + Radiomics combination led to a C-index of 0.786, which was lower than the Deep-Features + Radiomics combination, meaning that we could achieve accurate predictions of patients’ survival days without employing clinical data. Table 11 also demonstrated that the features based on radiomics and DeepFeat achieved the highest C-index scores (0.821) compared to those of methods that were specially designed for the prediction of patients’ survival days, such as [38,39,40,41].

## 4. Discussion

Image segmentation is the first stage in the volumetric quantification of the developing fetal brain, which is used to examine the neurological growth of fetuses with congenital abnormalities and inform prenatal planning. The predicted delivery date is crucial for an accurate estimation of gestational age when managing any pregnancy. The timing of appropriate obstetric treatment and the scheduling and interpretation of some antepartum diagnostics that assess the appropriateness of fetal growth and measures of development in order to prevent preterm births and associated morbidities depend heavily on accurate knowledge of the gestational age. We must create an automated, accurate, and precise procedure for fetal brain segmentation and gestational age calculation.

This paper tackled the tasks of fetal tissue segmentation and gestational age prediction. The 2D-based multi-view (axial, coronal, and sagittal) models were analyzed for fetal brain tissue segmentation. The end-to-end fetal tissue segmentation and GA prediction models were trained and tested with the FeTA 2021 MRI dataset. Initially, we trained our segmentation model on only axial slices. However, the results were not convincing, especially in brain tissues in which the number of class pixels was small. In addition, we acquired all axial, sagittal, and coronal slices from the 3D fetal brain input volume. We trained the model on those particular view slices. The coronal view model performed better than the sagittal and axial models based on the experimental results and observations. Later, the fusion of the predictions of the axial, sagittal, and coronal views were combined to enhance the model’s outcome.

Various regression techniques were developed for GA prediction by using different feature extraction methods, including a 3D autoencoder, radiomics features, and features extracted from the IRMMNET encoder. The IRMMNET encoder’s features were used with different regressors (LR, RF, RT, and XGB) to predict gestational age. Radiomics- and 3D-autoencoder-based features were also used for GA prediction. Extensive experiments were performed to achieve the optimal performance in optimal segmentation and gestational age prediction. Metrics such as those of the Kaplan–Meier and the SHAP explainability methods were used to study the explainability of the proposed GA models. Different comparisons and datasets were utilized to validate the generalization of the proposed models.

The main limitation of our study is that the sample size for each GA period in the held-out validation dataset was relatively small. Therefore, validating the proposed GA estimation model with data from larger populations and settings will be critical in order to extend the current use of this MRI-based biometric measurement prediction model to clinical application scenarios. Since fetal growth is influenced by each mother’s previous gestational history, body condition, and composition, future studies should consider mothers’ demographics as variables or covariables in their models, which might be useful for improving the precision of gestational dating. We chose multi-view multi-scale deep learning segmentation models in order to deliver the complete volumetric information and to provide an efficient application to fetal brain segmentation, gestational age prediction, and head and neck tumor segmentation and survival analysis. Our proposed approach consisted of various modules for predicting 3D segmentation maps with a limited dataset and lower computational resources. Our proposed approach produced a more efficient and faster response on the validation dataset for the task of fetal brain segmentation. The imaging features might not be sufficient to accurately predict gestational age. However, we used various feature extraction approaches, which included imaging, radiomics, 3D latent space autoencoder-based features, and deep features extracted from the last layer of multi-view 2D image slices from segmented brain tissues, to extract more localized features for gestational age prediction. The fusion of multi-scale segment-based deep features achieved better performance than that of state-of-the-art methods. There is a further need to investigate different methods with deep learning models for GA prediction. The existing methods are based on single-feature extraction techniques that use basic deep learning models. Correspondingly, the datasets used in existing methods are in-house and private. There is a need to set a benchmark on a publicly available dataset for further comparisons and enhancements in deep learning/machine learning for gestational age prediction and fetal brain segmentation.

We chose multi-view multi-scale deep learning segmentation models in order to deliver the complete volumetric information and to provide an efficient application to fetal brain segmentation, gestational age prediction, and head and neck tumor segmentation and survival analysis. Our proposed approach consisted of various modules for predicting 3D segmentation maps with a limited dataset and lower computational resources. Our proposed approach produced a more efficient and faster response on the validation dataset for the task of fetal brain segmentation. We developed a couple of imaging-based features and validated them on two different medical imaging datasets for segmentation, fetal age prediction, and head and neck survival analysis. This is a comprehensive end-to-end solution for fetal brain segmentation and gestational age prediction. Three types of features—radiomics features, clinical features, and latent features from the 3D autoencoder—were used for the prediction of gestational age with various regression models. These regression models were applied together with various feature fusion combinations for the prediction of gestation age. Our proposed model was also used in head and neck cancer segmentation. The head and neck segmentation features were used in the prediction of the survival age, and the performance of the proposed model was compared with the performance of state-of-the-art models for the tasks of head and neck cancer segmentation and survival prediction. We developed a couple of imaging-based features and validated them on two different medical imaging datasets for segmentation, fetal age prediction, and head and neck survival analysis. This is a comprehensive end-to-end solution for fetal brain segmentation and gestational age prediction. Three types of features—radiomics features, clinical features, and latent features from the 3D autoencoder—were used for the prediction of gestational age with various regression models. These regression models were applied together with various feature fusion combinations for the prediction of gestation age. Our proposed model was also used in head and neck cancer segmentation. The head and neck segmentation features were used in the prediction of the survival age, and the performance of the proposed model was compared with the performance of state-of-the-art models for the tasks of head and neck cancer segmentation and survival prediction.

## 5. Conclusions and Future Work

This paper proposed an end-to-end, fully automated, and effective method for multi-tissue fetal brain segmentation called IRMMNET. It is a multi-view segmentation model with significantly fewer parameters thanks to the inception residual encoder block (EB) and the dense spatial attention (DSAM) block, making it easier to extract information from multi-view MRI images that is pertinent to multi-scale fetal brain tissue and to improve feature reuse. In addition, this paper presented three approaches to the estimation of gestational age (GA). The first approach was based on a 3D autoencoder, the second approach was based on radiomics features, and the third approach was based on the IRMMNET segmentation model’s encoder.

Extensive experiments and analyses were provided, and these included the study of different configurations of the proposed segmentation model and the analysis of the impacts of various loss functions. We also examined the effect of applying the proposed fusion technique to other existing segmentation models, such as the UNet, ResUNet, and SE-ResUNet models. We found that the proposed fetal brain segmentation model obtained the best results with the Combo loss function, achieving a DSC score of 0.791 and an HD95 score of 21.66, outperforming other models. In addition, the density plot analysis demonstrated that with the proposed segmentation model, the distribution scores of all classes were greater than those with other models.

In the GA estimation task, different regression techniques (LR, RF, RT, and XGB) were assessed in combination with three feature extraction approaches (3D autoencoder, radiomics, and IRMMNET encoder). We found that when used with with RF, the radiomics features led to the best GA prediction results, with an RMSE score of 1.42 and a C-index score of 0.888. In addition, the analysis of the Kaplan–Meier plots proved the efficacy of the proposed GA prediction method based on the radiomics features and RF. Further, we studied the explainability and the importance of the radiomics features, and we found that the max 3D diameter and minor axis length were the most important features in GA prediction in comparison with the other features.

Finally, we studied the generalization capabilities of the proposed fetal brain segmentation and GA prediction methods for two tasks, namely, head and neck tumor segmentation and the prediction of patients’ survival days. When applied to head and neck tumor segmentation, we found that the proposed segmentation model outperformed existing models that were specifically designed for this task. In addition, we found that the proposed features based on radiomics and deep features extracted from the IRMMNET encoder achieved higher C-index scores (0.821) than those of the methods that were specially designed for the prediction of patients’ survival days.

The presented approach to segmenting the fetal brain and estimating gestational age and delivery dates could be utilized to accurately quantitatively analyze the human fetal brain and to determine the gestational age and expected delivery date, both of which are crucial for management decisions in any pregnancy. For the timing of appropriate obstetric care, scheduling, and the interpretation of several antepartum diagnostics, precise information on the gestational age is essential.

In future work, we will use different patch-based 3D segmentation models and 3D transformers for the task of fetal brain segmentation in order to further enhance the system’s performance. In addition, we will validate the GA estimation method with data from larger populations and settings.

## Figures and Tables

**Table 1:** Performance of the proposed segmentation model with the axial, sagittal, and coronal views.

Model	DSC	HD95	Sensitivity	Specificity
IRMMNET_Axial	0.778	24.06	0.8126	0.974
IRMMNET _Sagittal	0.781	22.80	0.817	0.974
IRMMNET _Coronal	0.789	21.56	0.818	0.976

**Table 2:** Comparing IRMMNET with different segmentation models.

Model	DSC (%)	HD95 (mm${}^{3}$)	Sensitivity (%)	Specificity (%)
BaseUNet-Axial	0.727	29.401	0.806	0.952
BaseUNet-Sagittal	0.723	31.381	0.790	0.947
BaseUNet-Coronal	0.728	29.042	0.813	0.963
ResUNet-Axial	0.748	26.111	0.827	0.968
ResUNet-Sagittal	0.756	27.334	0.818	0.969
ResUNet-Coronal	0.752	26.014	0.828	0.977
SE-ResUNet-Axial	0.762	28.262	0.809	0.973
SE-ResUNet-Sagittal	0.769	28.888	0.808	0.978
SE-ResUNet-Coronal	0.773	27.101	0.879	0.969
IRMMNET-Axial	0.778	24.062	0.819	0.972
IRMMNET-Sagittal	0.781	22.801	0.817	0.974
IRMMNET-Coronal	0.789	21.565	0.818	0.976

**Table 3:** Performance of various configurations of the proposed IRMMNET model.

Model	DSC	HD95	Sensitivity	Specificity
UNet	0.733	28.58	0.817	0.968
Multi-view_2D Inception + Residual	0.778	25.42	0.8178	0.967
Multi-view_2D Inception + Residual + DSAM	0.783	23.26	0.8101	0.976
IRMMNET	0.791	21.66	0.819	0.980

**Table 4:** Performance of IRMMNET with different loss functions.

Loss Function	DSC (%)	HD95 (mm${}^{3}$)	Sensitivity (%)	Specificity (%)
BCE	0.789	23.88	0.671	0.812
Dice	0.776	24.51	0.668	0.795
BCE + Dice	0.780	22.83	0.682	0.809
Combo	0.791	21.66	0.691	0.818

**Table 5:** Estimations of the training and validation times for the proposed and state-of-the-art methods.

Segmentation Models	Training Time (min)	Validation Time (min)
BaseUNet-Axial	45	2
BaseUNet-Sagittal	50	2.1
BaseUNet-Coronal	55	2.01
ResUNet-Axial	60	2.23
ResUNet-Sagittal	62	2.45
ResUNet-Coronal	61	2.11
SE-ResUNet-Axial	63	2.53
SE-ResUNet-Sagittal	65	1.95
SE-ResUNet-Coronal	64	1.88
IRMMNET-Axial	53	1.3
IRMMNET-Sagittal	55	1.4
IRMMNET-Coronal	52	1.5

**Table 6:** Comparison of the statistical analyses of the proposed IRMMNET and the state-of-the-art methods.

Segmentation Models	*p*-Value
BaseUNet-Axial	0.65
BaseUNet-Sagittal	0.68
BaseUNet-Coronal	0.72
ResUNet-Axial	0.89
ResUNet-Sagittal	0.87
ResUNet-Coronal	0.97
SE-ResUNet-Axial	1.15
SE-ResUNet-Sagittal	1.10
SE-ResUNet-Coronal	1.01
IRMMNET-Axial	1.63
IRMMNET-Sagittal	1.7
IRMMNET-Coronal	1.9

**Table 7:** Comparing the segmentation results of the proposed Multi-view-IRMMNET, Multi-view-ResUNET, and Multi-view-SE-ResUNET models.

Model	Dice	HD95	Sensitivity	Specificity
Multi-view_ResUNET	0.758	27.39	0.826	0.97
Multi-view_SE-ResUNET	0.772	27.56	0.812	0.973
Multi-view_ IRMMNET	0.791	21.66	0.819	0.980

**Table 8:** Comparison of the performance of the proposed Multi-view-IRMMNET model with that of two existing methods—DA_FaBiAN_Baseline and TopoCP (2D).

Model	DSC (%)
DA_FaBiAN_Baseline [34]	0.70 ± 0.24
TopoCP (2D) [35]	0.70 ± 0.14
Multi-view-IRMMNET	0.791± 0.18

**Table 9:** Analyzing the performance of different feature types and regression techniques for GA prediction.

Models	LR		XGB		RF		RT	
	RMSE	C-Index	RMSE	C-Index	RMSE	C-Index	RMSE	C-Index
Radiomics	1.70	0.837	1.477	0.854	1.42	0.888	1.44	0.858
IRMMNET Deepfeat	4.56	0.465	4.10	0.252	3.46	0.371	5.97	0.542
3D deep autoencoder features	4.51	0.418	3.71	0.427	3.26	0.512	4.64	0.517

**Table 10:** Analysis of the generalization capabilities of the proposed fetal brain segmentation model on the Hecktor 2021 dataset.

Method	DSC (%)	HD95 (mm)
IRMMNET	0.77	3.02
[37]	0.65	4.07
[38]	0.75	3.27

**Table 11:** Analysis of the generalization capabilities of the proposed feature extraction methods on the Hecktor 2021 dataset for the prediction of patients’ survival days.

Method	C-Index
Clinical features	0.692
Radiomics	0.791
DeepFeat	0.771
3D deep autoencoder features	0.723
Deep-Features + Radiomics	0.821
Clinical + DeepFeat + Radiomics	0.786
[38]	0.810
[39]	0.470
[40]	0.690

## Data Availability

The datasets used in this study are publicly available at https://feta.grand-challenge.org/feta-2021/. The source codes of the proposed method will be available at https://github.com/Moona-Mazher/Fetal-Segmentation-Gestational-Age-Prediction-Deep-Learning on 20 November 2022.
